# Organization of primary care

**DOI:** 10.1017/S1463423622000275

**Published:** 2022-09-01

**Authors:** Mehmet Akman, Duygu Ayhan Başer, Bugu Usanma Koban, Tino Marti, Peter Decat, Yann Lefeuvre, Robin Miller

**Affiliations:** 1 Marmara University School of Medicine, Department of Family Medicine, Istanbul, Turkey; 2 Hacettepe University School of Medicine, Department of Family Medicine, Ankara, Turkey; 3 Hilmi Şahin Aile Sağlığı Merkezi, İstanbul, Turkey; 4 Center for Research in Health and Economics, University Pompeu Fabra, Barcelona, Spain; 5 Ghent University, Department of Public Health and Primary Care, Ghent, Belgium; 6 Royal Arsenal Medical Center, London, UK; 7 Birmingham University Global Engagement for College of Social Sciences, Edgbaston, UK

**Keywords:** health policy, service organization, integrated care, accessibility, renumeration

## Abstract

Strong primary care does not develop spontaneously but requires a well-developed organizational planning between levels of care. Primary care-oriented health systems are required to effectively tackle unmet health needs of the population, and efficient primary care organization (PCO) is crucial for this aim. Via strong primary care, health delivery, health outcomes, equity, and health security could be improved. There are several theoretical models on how primary care can be organized. In this position paper, the key aspects and benchmarks of PCO will be explored based on previously mentioned frameworks and domains. The aim of this position paper is to assist primary care providers, policymakers, and researchers by discussing the current context of PCO and providing guidance for implementation, development, and evaluation of it in a particular setting. The conceptual map of this paper consists of structural and process (PC service organization) domains and is adapted from frameworks described in literature and World Health Organization resources. Evidence we have gathered for this paper shows that for establishing a strong PCO, it is crucial to ensure accessible, continuous, person-centered, community-oriented, coordinated, and integrated primary care services provided by competent and socially accountable multiprofessional teams working in a setting where clear policy documents exist, adequate funding is available, and primary care is managed by dedicated units.

## Introduction

The demonstrated links of primary health care to better health outcomes, cost-efficiency, accessibility, and enhanced health safety make primary health care the cornerstone of health systems strengthening (Kringos, 2015). Health systems built on the basis of primary health care are fundamental to succeed universal health coverage (WHO and UNICEF, 2020). Well-organized primary healthcare services can play a basic role in improving population health as well as the well-being of the population (Starfield, 2011).

Although significant improvements have been realized in the health outcomes of the global population during the era of the Millennium Development Goals, nearly half of the population cannot access the health services they need. This situation causes inequality in health. Health is central to the 2030 Agenda for Sustainable Development as it relates to many of the Sustainable Development Goals and is the specific focus of Goal 3 (WHO, 2015; WHO and UNICEF, 2020).

**Figure 1.**
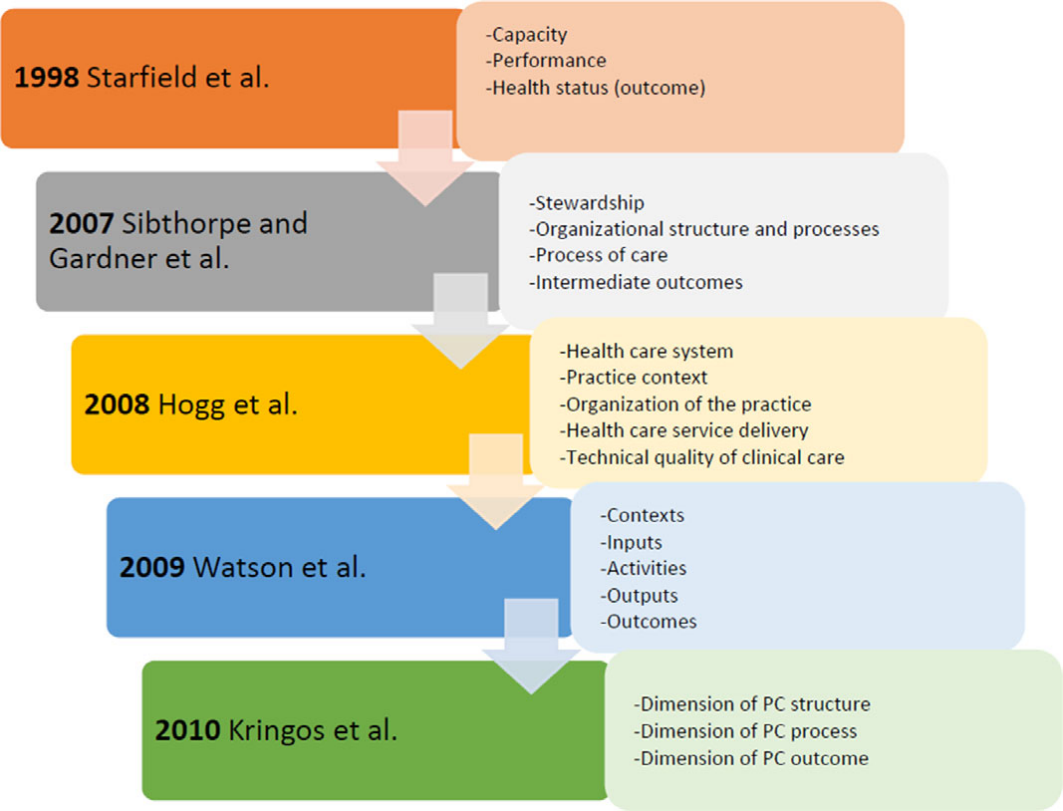
Historical mapping of the major frameworks on PCO

Several theoretical models have been developed in organization of primary care (Starfield, 1998; Sibthorpe and Gardner, 2007; Pineault *et al.,* 2009; Watson *et al.,* 2009; Hogg, 2008; Kringos *et al.,* 2010; Ebert, 2017). Defined frameworks having heterogeneous purposes are given in Figure 1. This figure gives an outline of the proposed frameworks on a timeline.

Barbara Starfield was the first author who started to explore the context of primary care organization (PCO). She mentioned that organization of primary care includes four main primary care domains: first contact care, comprehensiveness, continuity, and coordination (Starfield, 1979; Starfield, 1994). In 2007, the Australian framework was published, and it aimed to provide a performance assessment in primary health care via general overview of healthcare measurements (Sibthorpe and Gardner, 2007). One year later, Hogg *et al.* proposed that the conceptual framework for PCO consists of structural and performance domains, and this domain classification influenced new authors of the subject until today. The structural domain includes three components: the healthcare system, the practice context, and organization of the practice, and the performance domain includes two components: healthcare service delivery and technical quality of clinical care (Hogg, 2008). Watson *et al.* have drawn a broader framework and mainly focused on performance outcomes described as direct, intermediate, and final outcomes. They proposed a structure composed of inputs, activities, output, and outcomes (Watson *et al.*, 2009). Kringos *et al.* mentioned that there are diversities between countries’ PCOs as a result of different policy priorities (Kringos *et al.* 2013a). According to them, the structure of primary care consists of three dimensions: primary care governance; financing of primary care; and primary care workforce development. They determined primary care process by four dimensions: accessibility of primary care; comprehensiveness of primary care; continuity of primary care; and coordination of primary care (Kringos *et al.,* 2013b). In countries with strong primary care, the domains of this framework appear to be fully implemented (Kringos, 2015).

**Figure 2.**
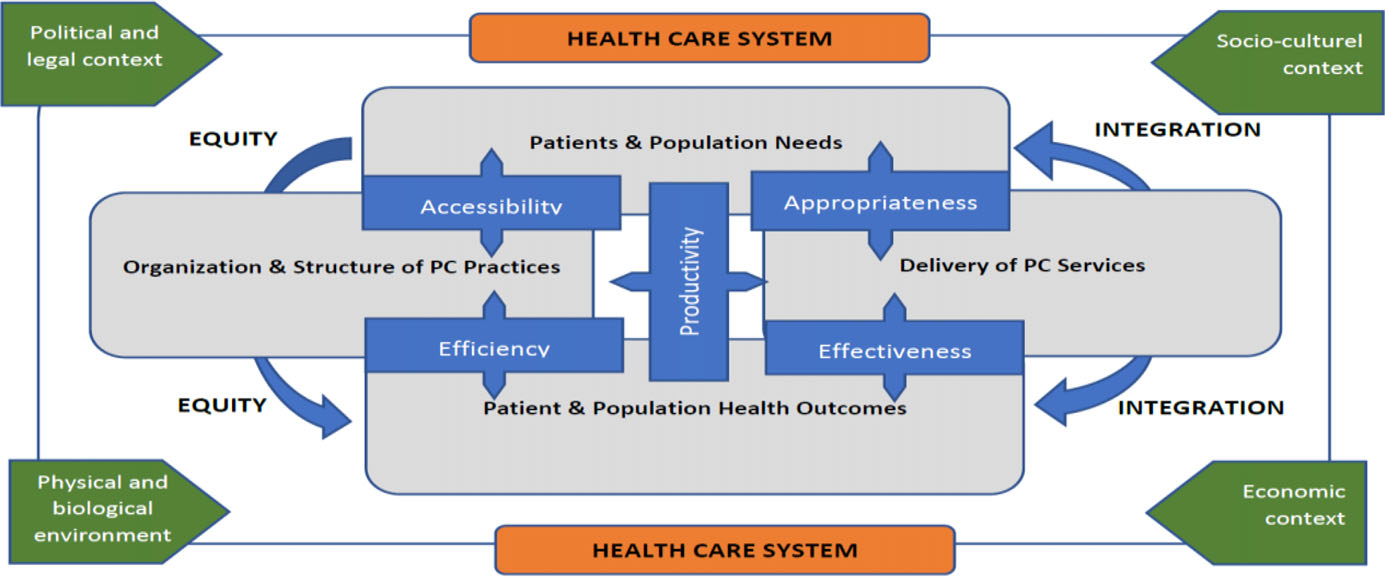
Consolidated framework for primary care organization

Senn *et al.* reviewed existing literature regarding PCO frameworks, and they proposed a consolidated framework as an end product of synthesis of existing information (Figure 2). Their framework is particularly beneficial for PCO designing and implementing well-defined monitoring activities (Senn *et al.*, 2021). With the addition of contexts such as socio-cultural, economical, and biological, the framework became multidimensional and in depth. Inclusion of needs and outcomes of patients and the population has a potential to cover productivity of the given organization. Also, having a consolidated framework providing a more comprehensive and uniform vision of PCO will enable international comparisons.

Outbreaks of global pandemics or emergencies like COVID-19 are the biggest challenges for the ‘resilience’ of primary care systems. Therefore, countries need to establish a regular system of facility assessments to provide objective measures for evaluating the availability, readiness, quality, and safety of health services, including measures to evaluate preparedness and response capacities (WHO and UNICEF, 2020). Even before the pandemic in 2017, the European Commission requested its Expert Panel on ‘Effective Ways of Investing in Health’, to provide an overview of domains and dimensions to be taken into consideration in assessing primary care and to give a better understanding of primary care performance. The Expert Panel described the scope of PCO and drew on the main characteristics of primary care and identified ten domains of primary care (classified by structure, process, and outcome). Inclusion of accountability as a dimension of PCO in this document is especially important to underline the need of a formal link between a group of providers and a defined population (Kringos *et al.*, 2019). The identified domains supply an origin for strengthening the PCO across countries.

Strong primary care requires a well-developed organizational planning between levels of care. Primary care-oriented health systems are required to effectively handle unmet health needs of the population, and efficient PCO is very important for the achievement of a primary care-oriented health system. In this position paper, the key aspects and benchmarks of PCO will be explored based on previously mentioned frameworks and domains. The aim of this position paper is to assist primary care providers, policymakers, and researchers by discussing the current context of PCO and providing guidance for implementation, development, and evaluation of it in a particular setting.

## Methods

**Figure 3.**
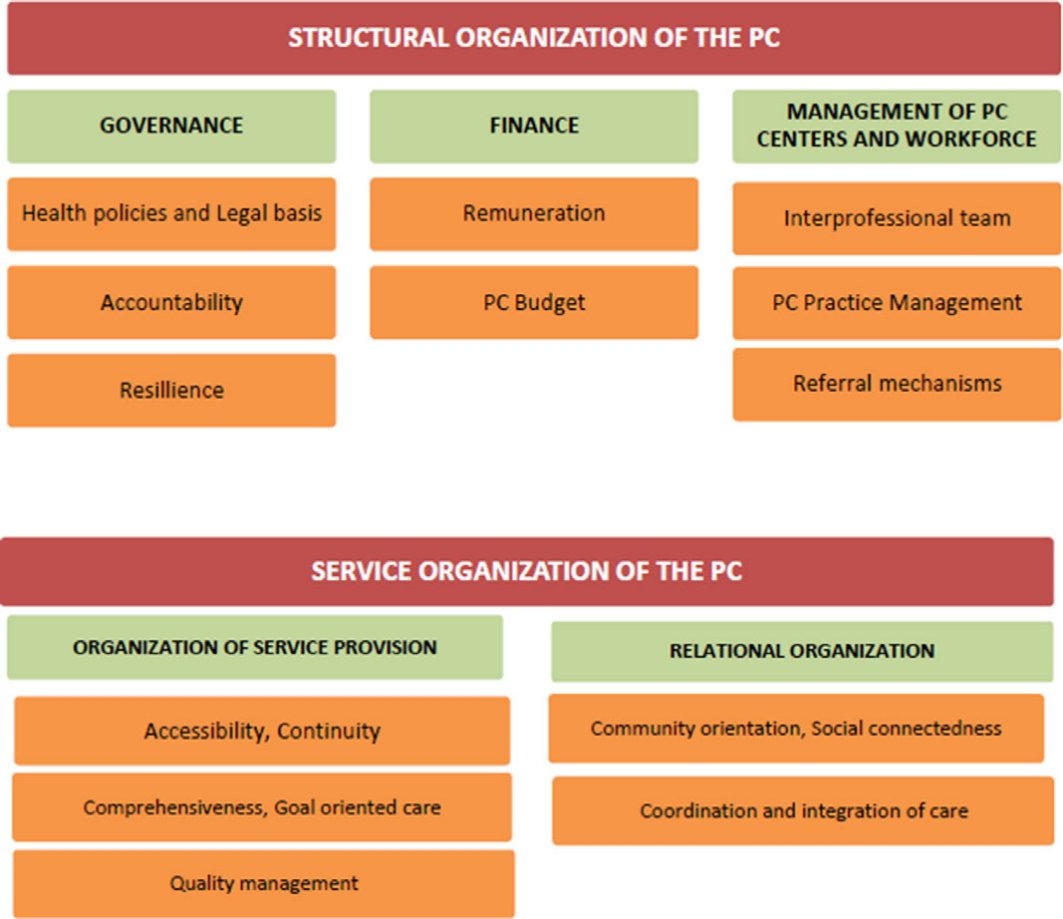
Structure and process levels of primary care organization: conceptual map

The conceptual map of this paper consists of structural and process (PC service organization) domains (Figure 3). For the adaptation of this framework, firstly the theoretical frameworks about PCO were explored and the concepts for this position paper were determined. The European framework (Kringos *et al.,* 2013b) was selected as the major theoretical background document as it is the one most cited in current literature. Secondly, domains described by the Expert Panel on ‘Effective Ways of Investing in Health’ for assessment of PCO and levels described in WHO operational framework for primary health care (WHO, 2020a) were synthesized and merged into the conceptual map of this position paper. Each concept in the conceptual map was assigned to a co-author, and each co-author prepared a text of 500 words.

Three authors (MA, DAB, BK) did several literature searches to be able to link information to scientific research and/or to available policy information. As a literature search strategy, relevant papers were searched from 2010 and onwards. Subject headings in the conceptual map were determined as keywords. The same three authors merged the texts provided by co-authors and organized the draft version. Then, the draft was opened to discussion to all co-authors. Necessary revisions were made according to feedback with consensus, and the paper is finalized.

Data gained by semi-structured European Forum for Primary Care (EFPC) workshops (2019 in Nanterre and 2020 online) were also used. These workshops allowed us to consult with international attendees, practitioners, and patients worldwide. The Nanterre workshop enabled the authors of this position paper to meet face to face and explore the content together. PIE (problems, ideas, experiences) digital membership and discussion platform of EFPC were used as a tool for online discussions and development of ideas and problems to be addressed (EFPC, 2018). On this platform, a theme called ‘Organization of Primary Care’ was launched in 2019. Since then, 10 new topics started and 69 shares under these topics were published. All relevant contributions were included in the development process of this paper together with knowledge obtained from experts during the EFPC conferences.

## Structural organization of the primary care

### Governance

#### Health policies and legal basis

Strong primary care needs a multi-level and intersectoral management to address its political, cultural, social, environmental, and economical components. Furthermore, an approach with enhanced horizontal and vertical relationship between governance levels and a synergistic perspective across sectors, institutions, communities, cities, and even countries is of great importance for an equal and comprehensive primary care service (WHO, 2020b). Especially, governance functions (eg, goals, policy-making, and definition of financing and regulatory mechanisms) impact primary care performance at practice and provider level, and according to up-to-date studies, its effectiveness is related to decentralization and financial regulation (Espinosa-González *et al*., 2019).

All practices, including primary care, should have well-developed organizational and clinical systems in place documented by their policies, protocols, and procedures. In addition, developing policy documents in primary care practice is the major indicator in The European Primary Care Monitor which was developed by Kringos *et al*. (2015). A vision on primary care should be clearly laid down in policy documents by government or important stakeholders. Also, a dedicated department or unit is required for a strong primary care within the health management scheme.

In a technical paper on governance of health published by WHO in 2020, four strategic levers for primary care are mentioned; these are political commitment and leadership, governance and policy frameworks, funding and allocation of resources, and the engagement of communities and other stakeholders. Actions and interventions related to all levers, in particular those related to governance and finance, need to be developed using an inclusive and ongoing policy dialogue that engages the community as an actor. For the balanced functionality of these levers, the scope of the existing policy should be increased, and the community should be organized as an actor (WHO, 2020b). In order for this organization to be carried out in the best way, the management should be transformed into a guidance position by shedding its role as a service provider. Although this adaptation will develop slowly, especially in low- and middle-income countries, it is important for inclusive and effective primary care services.

As a result of the data accumulated over the years in the literature, it has been seen that besides its successes the public governance system also has limitations like shortage of human resources, inefficient institutional frameworks, inadequate quality, and efficiency due to a lack of competition (Joudyian *et al*., 2021). In addition, current studies on low- and middle-income countries have shown that well-managed multidisciplinary teams are successful with the engagement of the public and private sectors of the community-governed primary healthcare system (Bitton *et al*., 2019). Therefore, a whole-of-society approach can enable the government to work together with all other stakeholders for a common goal. By engaging the private sector, civil society, communities, and individuals, the whole-of-society approach can strengthen the resilience of communities to withstand threats to their health, security, and well-being (WHO, 2020b).

Recent experience with global COVID-19 pandemic showed that countries having a prepared pandemic plan and a strong primary care system did not necessarily experience lower COVID-19 mortality rates; what appears to make a difference is planned and prompt response, and the degree to which primary care is mobilized to respond. Effective primary care responses with smooth integration with public health measures lead to lower mortality from COVID-19 (Goodyear-Smith *et al*., 2020). This is an important lesson showing prompt emergency response, and public health integration should be taken into account as far as governance of primary care is concerned.

#### Accountability

Accountability in primary care is one of the main governance tools. WHO defines accountability as being answerable to someone for decisions and actions (WHO, 2020b). Accountability is a multidirectional concept within and between organizations; vertical accountability relationships typically exist between government and providers of PCOs and public; horizontal accountability relationships are seen commonly between primary care providers and public and associations (Aday and Andersen, 1974). Interrelations and interconnectedness of all these partners form the basis of accountability.

Accountability has five essential components: legal, financial, professional, political, and public. These five components affect the success of primary care outcome, goals, cost-effectiveness, and satisfaction of patient’s/healthcare professionals, and all these components need to be provided for PCO accountability (Aday and Andersen, 1974; Joseph and Phillips, 1984; Dewulf *et al*., 2013). To strengthen and improve the accountability in primary care, the ministry should be in constant consultation with all stakeholders in primary care. Developing clear and measurable goals (patient/provider satisfaction, resource utilization, access to care, etc.); policies and monitoring tools for accountability requirements is crucial. Government, professional bodies, and primary care providers should know their roles and responsibilities, and people should take up their role as a partner in health care for more accountable primary care (Aday and Andersen, 1974; Joseph and Phillips, 1984). In addition, primary care providers should be accountable in terms of their social role. Accountability mechanisms may vary across the delivery models. The core of ensuring accountability is to define it, guarantee the involvement of all the relevant stakeholders, be realistic, and monitor the job satisfaction of providers in primary care at a good level.

#### Resilience

Another aspect of the governance of a strong primary care is resilience. Resilience is a dynamic and flexible skill. Strong primary care systems must be able to adapt to changing environments and apply innovative solutions, so they need to be resilient. To establish a resilient primary care, stable funding mechanisms, risk assessment methods, good governance with well-defined responsibilities of skillful healthcare workers, building strong networks, ensuring sustainability are the essential elements (Ezekiel, 1996; Dowling *et al*., 2008). Besides the resilience of primary care as a system, individual provider’s resilience is also important. A successful primary care team leader is the one who could enable resilience in his/her primary care team. Stress management abilities, self-awareness, social competence, and reflective clinical practices are useful tools to improve primary care providers’ resilience (Brinkerhoff, 2004).

The recent pandemic of COVID-19 is testing the resilience of health systems, even in well-resourced countries. Its challenges have brought into focus the need to protect our global health with more sustainable primary care within a well-coordinated health system that has strong government and public support for its policies. However, to achieve this, primary care needs to have adequate resourcing and ability to strengthen its capacity to maintain essential services (Huston *et al*., 2020).

### Finance

#### Financing primary care: current and recurrent challenges

Primary care services are essential for the well-functioning of any health system regardless of its national configuration. Strong primary care systems provide through multidisciplinary teams a community orientation intertwined with public health and social services in addition to the first level of care that brings access to other levels of care. Multiple levers are needed to improve primary care as set forth by the renewed Declaration of Astana (WHO, 2018b) and instrumentalized through the Operational Framework (WHO, 2020b). Among the core strategic levers, funding and allocation of resources seeks to provide adequate funding allocated to promote equity in access, provide an incentive environment to enable high-quality care, and minimize financial hardship. Therefore, how we do fund primary care and reward health professionals becomes tantamount to strengthening not only the primary care sector but also the entire health system.

Looking at how primary care professionals are rewarded, diverse payment modalities have been experimented to incentivize primary care doctors and nurses, being the most used salary, capitation, fee for service, and pay-for-performance schemes. Each payment scheme introduces different incentives and disincentives to activity, quality, and performance that need to be balanced and adjusted in relation to the intended objectives. There is not an optimal payment model that stands out. Most health systems have opted for a mixed system combining salary and pay-for-performance or capitation and fee for services. For instance, the caveats on productivity brought by salary schemes can be compensated by pay-for-performance incentives that health organizations use to align primary care activities to health priorities.

From an aggregated perspective, the funding assigned to the primary care sector is also of vital importance and a significant signal to the health ecosystem of organizations and professionals. Despite the central and capital role, primary care has been often neglected from a resource allocation perspective due to diverse and historical reasons. In advanced primary care systems, this allocation ranges between 15 and 20% of the healthcare budget in contrast with more than 50% absorbed by hospitals. Initiatives like 30 by 2030 (De Maeseneer, 2020) appeal to raise the amount of resources allocated to primary care services up to 30% of the national healthcare expenditure aiming at achieving universal health coverage and a prevention-driven health and care system.

Against the backdrop of the current epidemiologic context, the role of primary care to respond effectively to the pandemic challenges is clearer. Through contact tracing, community action at care homes, or massive vaccination, while preserving services to non-COVID conditions, primary care performance has been uneven depending on its pre-crisis capacity. Bolstering primary care with more resources and better-adjusted payment systems to incentivize preventive activities and integration of care will remain as recurrent challenges for the years to come.

### Management of primary care centers and workforce

#### Interprofessional team

When we consider the definition of primary health care, agreed by WHO in the Declaration of Astana in 2018, it becomes obvious that primary care cannot be delivered effectively unless all parts of the primary care team can work together (WHO, 2018a). Strong, community-oriented primary care that meets the needs and health outcomes for patients, families, and communities in a context of Universal Health Coverage demands strong interprofessional working. The interprofessional team will vary in its constituents from one healthcare system to another (Freund *et al*., 2015), but can essentially be the general practitioner and nursing staff, and in the wider team includes physiotherapy, occupational therapy, midwife, community pharmacist, dentist, public health practitioner, physician’s assistant, social workers, and podiatrist. In some primary care settings, counseling, psychology, and mental health services are also offered. This array of expertise demands appropriate coordination and organization to meet the health needs of individual patients, and the community, in order to provide primary care that is integrated with other services. Interprofessional teams need support to collaborate well together. Each member of the team brings their own expertise, the skills, and competencies they have been prepared for in their professional training and their ability to contribute to the holistic care of the patient and their family, within the community. It seems incontrovertible that alongside the medical skill of diagnosis and prescribing of treatment, referral to secondary care, the skills of wound care, chronic disease management, pain management and rehabilitation, patient education and counseling and public health should be carefully planned and navigated for each patient care plan. Crucially, the skill mix should include the patients’ own expertise about their health, illness, and background experience as an essential component of integrated care. Patient involvement and participation in their own health care is now an accepted and expected element of the skill mix, especially with the wide availability of digital health and social media that support patients to develop their knowledge and make informed decisions.

There are studies and a few systematic reviews published but rarely have they attempted to assess cost-effectiveness in relation to coordination. A recent qualitative study in the United Kingdom compared three different practices introducing non-medical skill mix and concluded that it was a complex process and that *‘Recognition of factors affecting the assimilation of roles may help to better align them with the goals of general practice and harness the commitment of individual practices to enable role sustainability’* (Nelson *et al*., 2019). The study is based on 22 qualitative interviews and provides important evidence of process but cannot answer questions of cost against quality and health outcomes. As concluded by Williams *et al.,* there remains a significant need for well-designed studies of the effectiveness of skill mix in primary care (Williams *et al*., 2010).

Within this framework, there are still some important unanswered questions within current literature and policy documents. Some of these questions need to be answered with new data and research are ‘Does interprofessional working in primary care with a skill mix enhance equitable access to health care, who should coordinate and how is skill mix and sharing of tasks decided?’, ‘Should this be based on the demand-led (patient need) model of care or supply-led (availability and cost), how can demand and supply be balanced in relation to cost and quality of care in the light of significant workforce issues for general practitioners and nurses globally?’, ‘What contribution can patients and carers make to interprofessional working?’, ‘What evidence do we have of the cost-effectiveness of a well-coordinated skill mix in primary care that is acceptable to communities?’

#### Primary care practice management

##### Volume and duration of consultations, practice size, and type

What is the ideal practice size and type in primary care is a debated issue. Various studies have been published on the advantages and disadvantages of small and large practice size since the past years. According to these, there is an economy in terms of staff or employees and the use of information technologies in large practices (Ping and Ling, 2012). In small practices, it is suggested that patients find the health care more accessible and establish a closer relationship with the doctor and staff and thus have a higher patient satisfaction (Ng and Ng, 2013). Although the effect of practice size on certain age groups or specific medical conditions has been investigated in current studies, no clear impact on quality of care has been identified.

Similarly, there is no ideal method in terms of practice type. Although ambulatory consultations are widely practiced, home visits may also be preferred, especially in regions where elderly or home-dependent patients are predominant. In addition, telemedicine which is frequently used especially during current pandemic and virtual visits that include secure email and video engagement are also the types of practice discussed recently (Duffy and Lee, 2018; Lurie and Carr, 2018). However, in regions with low digital literacy or with technological limitations, the necessary infrastructure of these options should be built to provide equity in service. Primary care physicians also work in hospice services in many countries. Especially for conditions like pulmonary or cardiovascular diseases, infections, gastrointestinal diseases; metabolic or autoimmune disorders; and mental health issues as well as post-surgical or post-traumatic care needs; hospitalist procedures are beneficial (White and Glazier, 2011).

Although the average consultation time can be used as an outcome indicator for both the safe and cost-effective use of drugs for primary care and effective communication, there is no consensus on the ideal duration. In the review published by Irving et al, it was seen that the consultation time in 67 countries ranged from 48 sec to 22.5 min (Irving *et al*., 2017). According to the literature, short consultations are associated with more malpractice, more frequent visits and workload and more drug prescription (Irving *et al*., 2017; Petek *et al*., 2008; Valverde *et al.,* 2018). In contrast, longer consultations may be more effective in terms of patient satisfaction and patient-centered comprehensive care (Valverde *et al*., 2018; Gude *et al*., 2013).

##### Patient list size

Optimal panel size is an issue that can be evaluated differently from governmental, financial, and healthcare perspectives. Increased panel size may have negative effects on quality indicators such as accessibility, comprehensiveness, and patient satisfaction (Stefos *et al*., 2011; Dahrouge *et al*., 2012). In addition, having large patient lists makes service difficult for physicians working in urban areas or dealing with high-need patients (Muldoon *et al*., 2012). It may be beneficial to establish interprofessional teams in primary care in order to provide effective service to the expanding primary care service profile and increasing patient list size.

#### Referral mechanisms

Another important factor in the quality and continuity of the primary care service is the demand management. For an appropriate referral process, a whole system approach is needed because this intervention is the first part of the network between primary and secondary care (Blank *et al*., 2014). Generally, a whole system approach is about recognizing the various elements of a health system and evaluating the nature of the links and relationships between each of them (primary, secondary, and tertiary care). Recently, e-referral and e-consultation have been preferred among the various referral methods. Although it seems to increase the responsibility and workload, e-consultation is beneficial for the primary care physician to have information on specific issues as well as for the patient to receive effective service with minimal waiting time (Lee *et al*., 2018; Liddy *et al.*, 2016). In any case, it is possible for the patient to receive fast and effective care service, by providing accurate and comprehensive information exchange between the specialist and the primary care physician (O’Malley and Reschovsky, 2011).

## Service organization of the primary care

### Organization of service provision

#### Accessibility & Continuity

An essential feature of primary care is providing access to services for all who need them without financial hardship (Universal Health Coverage). Currently, at least half of the people in the world do not receive the health services they need because of out-of-pocket spending on health (WHO, 2015). Since primary care focuses not only on preventing and treating, in addition helping to improve well-being and quality of life, it is more important to have accessible primary care services for all, including vulnerable groups (especially, migrants, people with a limited social network and poor socioeconomic status or with learning disabilities, etc.)

To improve accessibility of primary care, volume and size of practice should be regulated according to regional features; the out of working hours accessibility should be increased; centers might be established close to the densely populated areas, phone access to the practice and after-hours access to clinicians via email, phone, or in-person visits should be improved and waiting times can be reduced (Kringos *et al*., 2012; Eklund *et al*., 2019; Moreira, 2018). In literature, it was mentioned that utilization, quality of primary care, cost-effectiveness, and patient satisfaction would all improve with better access to primary care (Glass *et al*., 2017; Kringos, 2015).

The modes of healthcare accessibility were changed during the COVID-19 pandemic, to reduce contagion; healthcare workers lead to conduct all consultations remotely (via e-consultations) unless there was urgent need otherwise. However, it will be very difficult to digitalize health in case of lack of equipment and low digital literacy of patients and it is hard to build up strong patient-health provider relationship via digital platforms only.

Continuity in care is a complex, multifaceted concept, with four domains: relational, longitudinal, informational, and management continuity. It is obvious that a healthcare service covering these four main domains of continuity will be successful in many aspects such as increased patient satisfaction, physician satisfaction, better physician-patient relationship, better adherence to medication, and reduced hospital admissions (Ionescu-Ittu *et al*., 2007; Freeman *et al*., 2003). The evidence from primary care studies suggests that improving continuity brings benefits both in terms of clinical outcomes and cost-effectiveness (Paul *et al*., 2013). Continuity of primary care should be facilitated by creating registered patient lists, by keeping records regularly, by organizing into small teams to care for a subset of the patients registered at the practices with large patient lists (Health Quality Ontario, 2011).

#### Comprehensiveness & goal-oriented care

More comprehensive primary care systems are associated with greater equity and efficacy in health care, reduced care fragmentation, spending and inpatient services utilization, better patient experience of care, and health outcomes (Royal College of General Practitioners, 2015; O’Malley and Rich, 2015). To promote high-quality comprehensive primary care services, health systems should strive to enhance the capacity and infrastructure of primary care, individuals and communities should be empowered to participate in health promotion. As a part of building comprehensive primary care systems, it is essential for primary care services to meet the complex needs and demands of the entire population. Multidisciplinary care teams can help to deliver comprehensive services for these complex needs of populations.

Person-centered primary care is beginning to form the cornerstone of high-quality health care. Person-centered care puts the person at the heart of their care, and in contrast to patient-centered care (visit-based), it is specifically focused on the whole person, and it also supports the collaboration and shared decision-making between person and physicians in management of care. Person-centered health systems engage people as equal partners in promoting and maintaining their health. To achieve patients’ maximum health potential while reaching toward individually defined goals, the goal-oriented care is a useful modality. Goal-oriented care relies on the patient’s desires and targets of achievement. However, in order to be empowered users of the health system and find appropriate ways to explore the ‘life goals’ of patients, patients must have the ability to make informed decisions, define their expectations/life goals, and participate in their own care (Health Quality Ontario, 2011). Health literacy is very important to help individuals become partners in co-production of health and also to involve in goal-oriented care (Royal College of General Practitioners, 2015).

#### Quality management

Quality in primary health care is a complex and multi-dimensional concept. Although the content of quality varies from country to country depending on many factors (demographics of the community, the geographic region, cultures, health systems, etc), a high-quality primary care is universally defined as ‘The Excellent Care for All Act’ as one that is accessible, appropriate, effective, efficient, equitable, continued, integrated, patient-centered, population health-focused, and safe (O’Malley and Rich, 2015). In other words, high-quality primary care is the cornerstone of universal health coverage.

Measuring quality in primary care is very complex, because primary care systems have many values, problems and have a very wide content. The measurements should cover the different aspects of quality, for example: Patient centeredness, access to, equity in and content of care, process and clinical outcome measurements and work satisfaction of physicians and other personnel. In recent years, the use of quality indicators has increased. Structure, process, and outcome aspects should all be part of such an assessment. Availability and accessibility of care, clinical competence, effectiveness, communication skills, interpersonal attributes, organization of care, including continuity and coordination of care are the different facets of primary care, and indicators can be developed for each of these aspects (EQuiP, 2017). Although quality indicators are useful as starting points for assessment process, many of the goals and values in primary care cannot be measured, for example, ethics and humanism in consultations, etc (EQuiP, 2017). In an umbrella review of primary healthcare quality indicators, 33 systematic reviews related to indicators were categorized according to the dimensions, funtion and type of care. Of a total of 727 indicators or groups of indicators, 74.5% were classified in the process category (eg, comorbid psychiatric conditions and response to treatment), followed by outcome (20.0%) (eg, potentially preventable hospitalization clinic indicator of chronic obstructive pulmonary disease) and structure (6.0%) (eg, availability: number of physicians per unit of populations) (Ramalho *et al*., 2019). Developing and regularly updating quality indicators for primary care within the framework of each country’s health system could be an appropriate step for quality control and improvement (Arvidsson *et al*., 2019; Ogundeji *et al.*, 2016; Ramalho *et al*., 2019; NICE, 2021).

### Relational organization

#### Community orientation, social connectedness

The COVID-19 crisis has highlighted that health systems should be oriented toward communities (Johnson and Goronga, 2020). On the one hand, community orientation is needed to make social interventions successful and sustainable. Health-promoting measures such as the current social distancing rules to prevent the spread of the epidemic or vaccination strategies are only effective if they are adopted and supported by a large part of the society. Therefore, social interventions should be understood and supported by the different echelons of the society. To achieve this, the involvement of communities in the development and implementation of health policies is essential. Failing to hear people’s concerns and to address them will lead to resistance.

On the other hand, community orientation is essential to reach people who are disproportionately affected by health threats. These vulnerable populations may include people with a limited social network and poor socioeconomic status, migrants, and minorities. Primary care providers often fail in reaching out to these vulnerable groups, and there is evidence that the community itself has a social capital that can contribute to accessing hard-to-reach and marginalized groups.

Also in the post-COVID-19 era, the involvement of the community in organizing health care will remain crucial for sustainable health policies that penetrate into the heart of the society (Perry, 2018; Sacks *et al*., 2020).

Health policies are often implemented in communities and not with the communities. Although health authorities frequently call for community engagement, haziness exists about how community orientation is taken into practice. What is needed to make primary care and primary care services effectively community-oriented?

Firstly, there is a need of awareness for accountability to the community among the primary care health workers. In general, health providers are motivated to meet the needs of individual patients. At that point, social prescribing is also recommended to help patients for acquiring healthy behaviors and better managing their health problems (Drinkwater *et al*., 2019). Conversely, primary care workers feel less comfortable in acting on a group level. This is a shortcoming in the training of the health workforce (Decat *et al*., 2019). Medical training institutes should make major efforts to improve the competencies of future caregivers to provide community-oriented care. The public health and primary healthcare integration should be done both at an educational and systemic levels. Place-based learning through participation at health-promoting and awareness-raising activities might be an effective strategy. The payment system of the healthcare workforce might be another limiting factor (Heider and Mang, 2020). For providers in a fee-for-service healthcare system, there is no incentive to work with the community as they are only paid for services provided to individual patients.

Secondly, there is a need for strategies on how communities can be actively involved as partners in the development and implementation of local health policies. European health systems may learn this from strategies used in low- and middle-income countries. Settings with limited access to basic health services have developed experience in engaging communities to reach underserved populations and to promote healthy behavior (Perry *et al*., 2017). For example, the mobilization of community health workers (CHWs) to support the health workforce in maternal and child care, in health promotion campaigns, or in epidemic control is common in Latin America, Africa, and Asia. Currently, there is growing evidence that CHW programs are effective to address health inequities in high-income countries (Javanparast *et al*., 2018).

The current pandemic shows the urgency for bringing communities at the center of primary care. We plead to set up an international thematic network on community-oriented primary care that operates as a learning community to collect, test, and adapt strategies for training the current and future health workforce in community orientation and for engaging communities as full partners in the organization and provision of local care.

#### Coordination and integration of care

Better coordination of care for patients and their families has long been seen as a core feature of good quality primary care (Kringos *et al*., 2012). Defined by the WHO as a proactive approach to bringing together care professionals and providers to meet the health needs of the population and to ensure that they receive integrated, person-focused care across various settings (WHO, 2018a). Care coordination’s importance has grown with the increasing rates of people experiencing chronic and multiple health conditions (EU Companion Report, 2017). Gaps in care coordination are associated with lower satisfaction for patients and families, unnecessary healthcare expenditure, and a higher risk of patients experiencing medical errors (Penm *et al*., 2017). Care coordination occurs at multiple levels – by the patient and the family themselves, within one organization (ie, multiple professionals within a single community health provider or general practice), and between multiple organizations through vertical (ie, sequential) and/or horizontal (ie, parallel) provider networks (Sheaff *et al*., 2015). Care coordination is a core component of case management which involves proactive case finding, assessment and care planning for at-risk populations. Such comprehensive programs can improve patient-centered care, facilitate self-management of long-term conditions, enable greater independence, and enhance patient satisfaction (Buja *et al*., 2020). The impact of selfcare management on healthcare expenditure and long-term survival rates is less clear, but greater in systems without a strong tradition of multidisciplinary primary care teams and where there is involvement of social workers alongside healthcare professionals (Stokes *et al*., 2015).

An important recent development for care coordination has been the recognition that traditional primary care services must better integrate with agencies that address people’s broader needs (National Academies of Sciences, 2019; Miller *et al*., 2016). This includes agencies that provide ‘social care’ services such as support with personal care (eg, washing and dressing), managing tasks of daily living (eg, shopping and cooking), and support for family carers (eg, respite breaks). Social care can be provided in a variety of settings including someone’s family home, designated supported housing schemes, and nursing homes with 24-hour care on site. Care coordination for particular populations encompasses services relating to the broader determinants of health and well-being including employment, education, domestic violence, and substance misuse. This can include access to charitable resources and voluntary support through social prescribing or community navigator schemes (Tew *et al*., 2019).

There is no-one approach to care coordination that works effectively in every context for all populations (Øvretveit, 2011). Achieving successful coordination is multi-factorial and requires planned system-level activity within and between organizations (Valentijn *et al*., 2015). Common enablers include defined referral pathways and processes, information agreements regarding patient assessment and plans, shared electronic records and technological facilitators, self-management training and support for patients and their families, and relevant quality management tools to improve coordination processes (Rijken *et al*., 2017; WHO, 2018a). For those with the most complex needs due to health or social circumstances, the identification of a named coordinator can be beneficial. The coordinator can provide continuity for the patient and their family, advocate for their interests in multi-agency meetings, ensure that there is good communication between professionals, and facilitate regular reviews of the care provided (Goodwin *et al*., 2013). Depending on the needs, workload pressures, and scope of their professional role, the coordinator may be an existing member of their multidisciplinary team who has an established relationship with the patient and their family (Penm *et al*., 2017). However, in other circumstances it is better for a professional who is introduced with the specific remit of coordination to ensure that they have the necessary capacity, connections, and authority to negotiate with other services. Professionals will need training and ongoing learning opportunities to ensure they develop and maintain the required competencies for coordination. This may be best achieved through interprofessional education to enable information sharing and experience of working closely with those from different disciplines (Miller *et al*., 2019).

## Conclusion

This position paper proposes a simplified and practical conceptual map for concepts related to PCO derived from synthesis of policy documents and theoretical frameworks. Authors have identified two main domains of structural and service organization. Under these domains, five dimensions of governance, finance, workforce and facility management, organization of service provision, and relational organization have been explored. Major key recommendations aiming to guide researchers, policy makers, and institutions of interest, were highlighted in box 1 and box 2.

Box 1. Key Recommendations for Structural Organization of PC

**Governance**

1. Clear policy documents and a dedicated department or unit are required for a strong PC within the health management scheme.
2. Actions and interventions for strengthening PC need to be developed with an engaged community as an actor.
3. Interrelations and interconnectedness of all stakeholders of PC form the basis of accountability.
4. The PC governance should include clear and measurable goals, policies, and monitoring tools.
5. PC providers should be accountable in terms of their social role.
6. Stable funding mechanisms, risk assessment methods, good governance, strong networks, ensuring sustainability are the essential elements for a resilient PC.

**Finance**

1. Adequate funding for PC is necessary to promote equity in access and minimize financial hardship.
2. Any incentive given to PC providers should enable high-quality care.
3. Up to 30% of the national healthcare expenditure is needed at achieving universal health coverage and a prevention-driven health and care system.

**Management of PC centers and workforce**

1. Strong and community-oriented PC is enhanced by multiprofessional teams.
2. Interprofessional working in primary care with a skill mix enhances equitable access to health care.
3. Establishing multiprofessional teams in PC might be useful for broadening service provision and to expand patient list size.
4. Home visits may be preferred in regions where elderly or home-dependent patients are predominant.
5. Telemedicine can be one of the appropriate ways for healthcare delivery in special conditions (during pandemic, etc.). The necessary infrastructure of telemedicine should be ensured without causing inequity in service provision.
6. E-consultation should be beneficial for the patient to receive effective service with minimal waiting time.
7. Referral systems are needed for demand management of health services. For an appropriate referral process, a whole system approach is needed.

Box 2. Key Recommendations for Service Organization of PC

**Service organization**

1. Accessibility can be improved by regulating the volume and size of practice, increasing the services during out of working hours, establishing centers close to the densely populated areas and reducing waiting times.
2. Continuity of primary care should be facilitated by creating a registered patient list and well-organized, integrated electronic health records.
3. Person-centered care should be the main focus of all patient encounters in primary care.
4. Health literacy should be improved to involve patients in person-centered care.
5. The quality measurements should cover patient centeredness, access to equity in and content of care, work satisfaction of primary care personnel.
6. Utilization of regularly updated quality indicators is needed for sustainability of high-quality primary care services.

**Relational organization**

1. The involvement of communities in the development and implementation of health policies is essential.
2. Place-based learning through participation at health-promoting and awareness-raising activities is an effective strategy for community orientation.
3. Engaging community health workers (CHWs) to communities is a good practice to reach underserved populations and to promote healthy behavior.
4. To achieve successful care coordination, referral pathways, and processes, information agreements should be defined, electronic records and technological facilitators should be shared, self-management training and support for patients should be provided.

PCO will continue to evolve to meet changing health needs of the populations as we have experienced during COVID-19 pandemic and will be shaped by good practices of primary care service provision supported by concrete evidence derived from primary care data. Hence, there is no model of organization that will fit in all settings in different countries, and implementation, development, and sustainability of an efficient PCO will remain as a challenge for all primary care-oriented health systems. For establishing a strong PCO, it is crucial to ensure accessible, continuous, person-centered, community-oriented, coordinated, and integrated primary care services provided by competent and socially accountable multiprofessional teams working in a setting where clear policy documents exist, adequate funding is available, and primary care is managed by dedicated units.
